# Small Hydropower Plants With Ecological Flow Influence Nestedness of Riverine Algae: Insights From treeNODF Analysis in Oujiang River Basin

**DOI:** 10.1002/ece3.72930

**Published:** 2026-01-12

**Authors:** Xinxin Qi, Zongwei Lin, Yuting Wang, Yuke Duan, Jiuli Shi, Huimin Gao, Sangar Khan, Naicheng Wu

**Affiliations:** ^1^ Department of Geography and Spatial Information Techniques Ningbo University Ningbo China; ^2^ Lianyungang Hean Middle School Lianyungang China; ^3^ Zhejiang‐Germany Joint Laboratory on Remote Sensing of Coastal Ecosystem Ningbo University Ningbo China; ^4^ Department of Geosciences and Geography University of Helsinki Helsinki Finland; ^5^ Department of Hydrology and Water Resources University of Kiel Kiel Germany

**Keywords:** ecological flow, periphyton, reservoir, small dams, stream, treeNODF

## Abstract

Understanding the nested distribution patterns is ecologically crucial for revealing river ecosystem heterogeneity and biodiversity maintenance. However, there is a notable research gap in understanding the nested patterns of river algae under the influence of small hydropower plants (SHPs), particularly in the context of ecological flow regulation. This study focused on the Oujiang river basin, where SHPs are widely distributed, exploring the effects of SHPs on the nestedness (NODF) of river algae under ecological flow and intensive development conditions. By calculating NODF, functional nestedness (traitNODF), phylogenetic nestedness (phyloNODF), and environmental nestedness (envNODF) for different river sections, the results indicated: (1) the entire basin and its different river sections exhibited significant nestedness; (2) the reservoir and dewatering sections were more strongly affected by SHPs, with traitNODF and phyloNODF remaining unchanged despite some environmental factors; (3) species with weaker attachment abilities and larger cell sizes tended to inhabit more unique habitats, particularly in reservoirs. These findings demonstrate that SHPs reshape algal communities primarily through environmental filtering, with reservoirs experiencing the strongest habitat modification. The study highlights the need for segment‐specific management strategies in SHPs development to balance water use and ecosystem conservation. Future work should further explore nestedness–ecosystem stability relationships under human disturbances.

## Graphical abstract

Algal nestedness is significantly present in river ecosystems affected by small hydropower plants (SHPs). Environmental filtering drives functional and phylogenetic nestedness in SHP rivers. Reservoir habitats show greater complexity in SHP‐affected rivers.

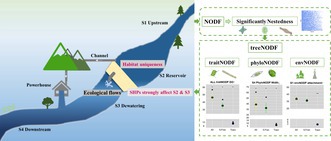

## 1 Introduction

Small hydropower plants (SHPs) are integral to advancing sustainable energy solutions and cutting greenhouse gas emissions, with over 80,000 plants operating worldwide by 2011 (Couto and Olden 2018; Kuriqi et al. 2021; Wu et al. 2007). In China, SHPs play a key role in reaching “carbon emission peaking” and “carbon neutrality” targets, particularly in rural and remote mountainous regions, where their low investment, minimal risk, and low operational costs make them crucial for providing electricity to over 1700 counties (Cheng et al. 2015; Fu et al. 2008; Gu et al. 2022). However, despite their importance in sustainable development and energy security, SHPs have raised significant environmental concerns, especially in sensitive mountainous regions that support unique habitats and endemic species. These areas are highly vulnerable to changes in river dynamics caused by SHPs, which can alter aquatic and riparian ecosystems, disrupt downstream flows, and lead to habitat fragmentation and ecosystem degradation (Alldredge and Moore 2014; Poff and Zimmerman 2010; Rood et al. 2010). Under intensive exploitation, some SHPs have reduced or eliminated ecological flows, leading to dewatering or decreased river flow during seasonal or dry periods, especially in diversion‐type hydropower stations (Wu et al. 2007). While some studies have explored the ecological impacts of SHPs, research on ecosystem effects under the context of ecological flow discharge remains limited (but see Lin et al. 2024; Qi, Lin, et al. 2024; Gao et al. 2025). Particularly, studies on the nested impacts of SHPs with ecological flows on different sections of significantly segmented rivers are scarce. Such research is crucial to identify specific sites and species, informing more scientifically based conservation strategies.

Nestedness, along with turnover, is universally recognized as one of the two fundamental components of beta diversity (Baselga 2010; Harrison et al. 1992). If the effects of nestedness and turnover on species distribution patterns are studied separately, it will be significantly important in the fields of ecology, biogeography, and conservation biology (Baselga 2010; Passy 2008; Potapova and Charles 2002). Among them, nestedness indicates the difference in species richness between communities (Baselga 2010; Legendre 2014; Podani 2016). However, there exists an extreme scenario where communities contain the same number of species with no overlapping species between them, resulting in differences driven purely by species turnover. In such cases, the nestedness calculated using multi‐site dissimilarity indices can still yield non‐zero results, which do not represent a strictly nested pattern (Almeida‐Neto et al. 2012). Therefore, this should be quantified using nestedness metrics, such as NODF (Nestedness metric based on Overlap and Decreasing Fill), which relies on pairwise overlap and matrix fill to provide a consistent assessment (Almeida‐Neto et al. 2008).

Nestedness has been extensively studied in the community of interacting species (Bascompte and Jordano 2007; Soininen 2008; Tornés and Ruhí 2013). It quantifies the overlap in species composition between high‐ and low‐diversity sites (Atmar and Patterson 1993), and measures the ordered nature of species gain and loss (Almeida‐Neto et al. 2008). Nestedness occurs when the species composition of species‐poor sites is a subset of species‐rich sites (Mitsuo et al. 2010). Since the nested pattern is an ordered arrangement of species distribution patterns, any factor that favors community order will increase the degree of nestedness, and vice versa (Baselga 2012). Among them, the major advantage of calculating nestedness using the NODF index lies in its intuitive interpretation and excellent statistical properties (it is unaffected by matrix size, shape, or fill; has a low Type I error rate; and is consistent with the definition of nesting). Furthermore, another feature of this index is that it not only calculates the total nestedness of the matrix but also allows for the calculation of the nestedness for individual sites or columns (NODFc) and for species or rows (NODFr). Since its introduction, NODF has been widely applied and is considered as the most suitable nested index currently available (Ulrich et al. 2009).

Nestedness can be defined as another scenario where a relatively large number of idiosyncratic species occupy subsets of the sites inhabited by more nested species (Almeida‐Neto et al. 2008). An important feature of nestedness is the degree to which different species contribute to perfect nestedness (Heino et al. 2009). Moreover, the characteristics of species determine their ability to colonize new habitats or their likelihood of extinction, and thus the ecological traits of specialist species may be related to their spatial structure (McAbendroth et al. 2005). Therefore, robust identification of nested structures in natural communities requires the collection of environmental data and functional trait data related to their constituent species (Ulrich et al. 2017). However, NODF operates at the species level, assuming that species are ecologically and phylogenetically equivalent (Wang et al. 2023). In reality, species in natural systems often play distinct roles in ecological functions and phylogenetic relationships (Petchey and Gaston 2002; Webb et al. 2002), necessitating the use of more suitable nestedness indices for quantification and analysis.

To address this issue, Melo et al. (2014) proposed treeNODF to calculate functional and phylogenetic nestedness in communities. The treeNODF is a novel nestedness index similar to NODF, but it constructs dendrograms to describe similarities among variables, replacing traditional species richness with branch lengths (Melo et al. 2014). Specifically, depending on the different approaches to constructing dendrograms, treeNODF can be divided into phylogenetic nestedness (phyloNODF) and functional nestedness (traitNODF) (Matthews et al. 2015). Concretely, phyloNODF uses the phylogenetic relationships (i.e., evolutionary relatedness) to build trees, thereby explaining phylogenetic diversity (PD), where the branch lengths represent PD (Chen et al. 2022). In contrast, traitNODF uses functional traits to construct trees, explaining functional diversity (FD), where the branch lengths represent FD (Melo et al. 2014). A key advantage of treeNODF is that it is not influenced by matrix size, fill, or tree topology (Melo et al. 2014). Similar to NODF, treeNODF can also be used to assess nestedness among communities (Chen et al. 2022). Furthermore, the framework can be extended to environmental nestedness (envNODF), which employs dendrograms describing the similarity of environmental conditions among sites to test whether the conditions for habitat‐specialist species are nested subsets of those for generalists (Melo et al. 2014). Importantly, treeNODF is a composite metric that can be further decomposed into the species composition component (S.Fraction) and the topological structure component (topoNODF) (Melo et al. 2014). If species are considered equally important in terms of ecological function and phylogenetic evolution, the observed treeNODF value corresponds to the S.Fraction value (Melo et al. 2014). The topoNODF represents the portion of treeNODF value driven by the topological structure of the tree, such as functional differences among species (Matthews et al. 2015). The decomposition of treeNODF helps to separately explore the relative importance of each component in shaping nestedness (Chen et al. 2022).

Research has demonstrated that examining nestedness across various biodiversity dimensions provides insights into species‐environment relationships, offering a more comprehensive understanding of community assembly processes compared to analyses limited to taxonomic composition alone (Almeida‐Gomes et al. 2019; Chen et al. 2022; Jacoboski et al. 2016). Nestedness is also generally considered beneficial for maintaining species diversity and ensuring the stability of network structure (Tornés and Ruhí 2013). It has been observed across various taxa in both terrestrial and aquatic environments, including plants (Honnay et al. 1999; Sasaki et al. 2012), birds (Cox and Blake 1991; Simberloff and Martin 1991), invertebrates (McAbendroth et al. 2005), insects (Schouten et al. 2007; Valdovinos et al. 2009), mammals (Fischer et al. 2010), macroinvertebrates (Ruhi et al. 2013), amphibians (Chen et al. 2022), and diatoms (Qi, Liu, et al. 2024; Soininen 2008; Tornés and Ruhí 2013). Currently, research on the nested pattern of diatoms has only been conducted in intermittent rivers in the Mediterranean (Tornés and Ruhí 2013) and several rivers in Finland (Soininen 2008) and China (Qi, Liu, et al. 2024). This research aimed to: (1) quantify the degree of nestedness in riverine algal communities under SHP influence across different river segments; (2) examine how traitNODF and phyloNODF nestedness respond to environmental gradients; and (3) assess how envNODF relates to species traits. We hypothesize that SHP‐induced habitat fragmentation and flow alteration will enhance overall community nestedness, particularly in reservoir and dewatering sections, and that multidimensional nestedness patterns will reflect environmental filtering mechanisms. This work provides an integrative framework for advancing understanding of algal metacommunity assembly in regulated rivers while delivering practical insights for segment‐specific management and ecological flow optimization in SHP‐affected watersheds.

## 2 Materials and Methods

### 2.1 Study Area

The Lishui city section of the Oujiang River Basin spans the area between Yuxi Hydropower Station and Kaitan Reservoir Dam (Wei et al. 2012). This section covers 316 km in length and a watershed area of 12,985.47 km^2^, representing 78% of Lishui's total area (Weng et al. 2017). Located in southwestern Zhejiang Province, Lishui city is characterized by a mountainous landscape with river basins, sloping from the southwest to the northeast, and experiences a central subtropical monsoon climate with significant mountain climate influences (Khan et al. 2025). These climatic and topographic conditions have endowed Lishui with abundant hydropower resources, earning it the title of “First City of Hydropower in China”. Additionally, Jingning County, under Lishui's jurisdiction, has been recognized as the “Township of Rural Hydropower in China”. By 2020, 799 SHPs had been established in the region.

**FIGURE 1:**
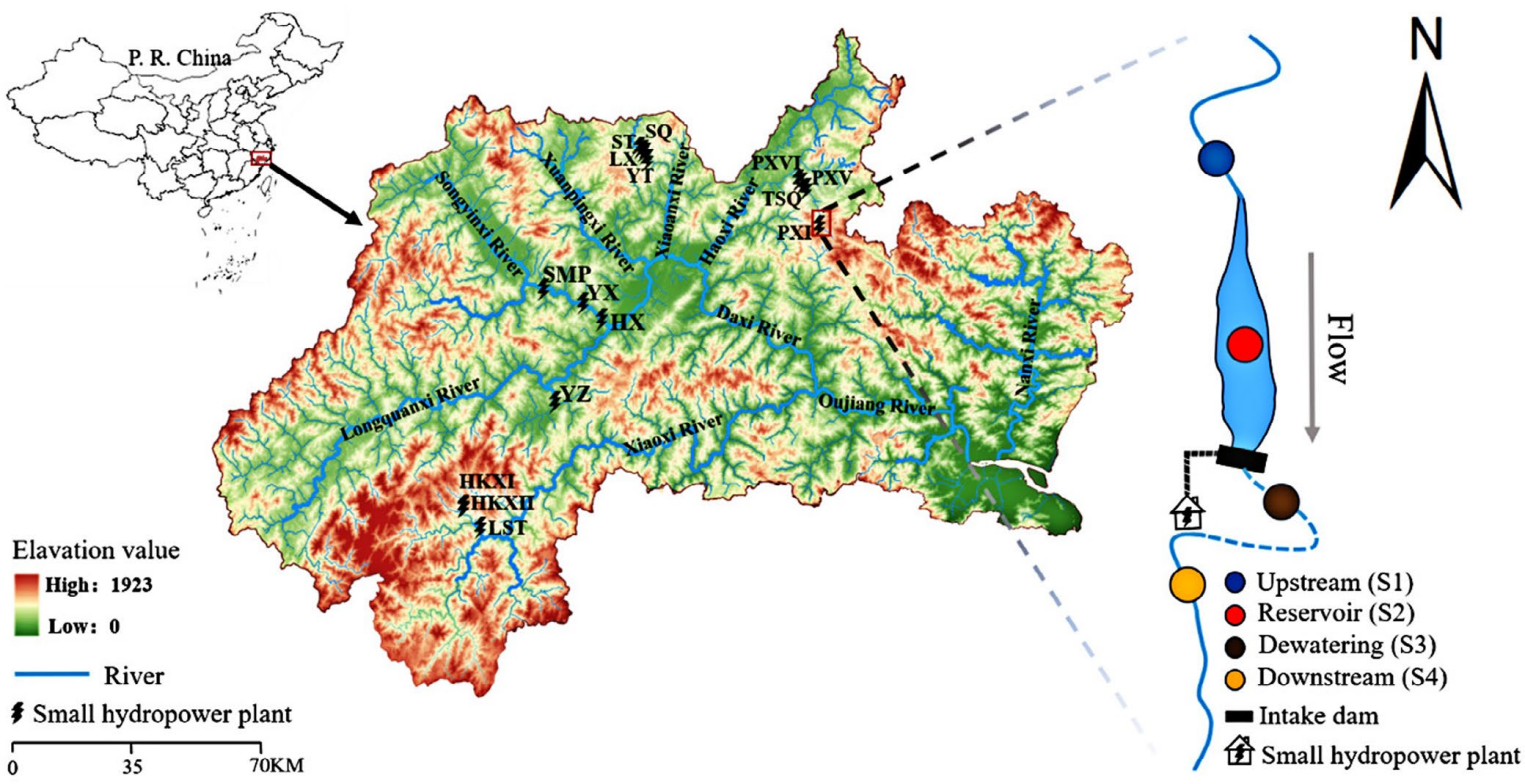
Distribution of SHPs in the Lishui section of the Oujiang river basin, China. Letters represent SHP abbreviations, for example, SMP indicates Shimapu SHP.

Based on the distribution of SHPs in Lishui and field observations, 15 representative SHPs were selected for aquatic biological surveys (Figure 1, Table S1). Following the classification framework of Couto and Olden (2018), most SHPs in the study area are diversion with storage type (e.g., TSQ, ST, LX), while a small number belong to non‐diversion with storage category (e.g., PXI, SMP). The ecological flow for non‐diversion with storage tends to be set at a relatively high level (e.g., SMP, YX, and HX). Four types of sampling sites were established for each SHP: Upstream (S1), Reservoir (S2), Dewatering (S3), and Downstream (S4). A total of 47 sampling sites were established based on their representativeness and accessibility. S1 was located in the upper reaches of the river, where the natural state remains largely unaffected by human activities. S2 was situated in the impoundment areas of the SHPs, and the corresponding depth data for these reservoir sections are provided (Table S1). S3 was positioned below the dam, where flowing water was maintained due to ecological flow releases. S4 (only for SHPs with diversion types) was located downstream, near the outlets of the SHPs or where diverted flows return to the main channel.

### 2.2 Field Sampling and Processing

The algae samples were collected in April 2023 from the river's littoral zone. Since the sampling sites of S2 were in the impoundment reservoirs of the SHPs, algae samples were collected by filtering a measured volume of river water through plankton nets (Wu et al. 2007). At other sampling sites (i.e., S1, S3, and S4), algae were gathered from natural substrates by selecting 3–5 stones from three distinct habitats per site. Each stone was carefully brushed, and the algae within a 4.8 cm diameter circle were rinsed with 380 mL of distilled water (Wu et al. 2009). The collected samples were preserved using 5% non‐acetic Lugol's iodine solution. Then, we examined the non‐diatom species in the 0.1 mL counting chamber under a microscope with 400× magnification (Wang, Wu, Tang, Wang, and Cai 2022). Next, permanent diatom slides were prepared in the laboratory using 30% H_2_O_2_ (Lane et al. 2003). Diatom valves were counted under oil‐immersion at 1000× magnification, with a minimum of 300 valves counted using the microscope. Species were identified to the lowest practical taxonomic level following the methodologies of Hu and Wei (2006), Malgorzata et al. (2012), and Maurice‐Yves (2013).

### 2.3 Environment Variables

Hydrological and physicochemical variables were collected at each sampling sites. We measured width (Width), depth (Depth) and flow velocity using GlobalWater flow meters (Tamodel BB1100, USA) and a rangefinder. Physicochemical variables, including water temperature (WT), dissolved oxygen (DO), conductivity (Cond), total dissolved solids (TDS), and pH, were measured using YSI (Model YSI‐6600V2, YSI Inc., Yellow Springs, Ohio, USA) handheld meter. Chlorophyll‐*a* (Chl*a*) was collected and analyzed following the Methods for Monitoring and Analysis of Water and Wastewater (State Environmental Protection Administration 2002).

### 2.4 Traits

Traits for each species were assigned using a binary coding system, encompassing three primary categories: cell size (nano, micro, meso, macro, or large), guild (low profile, high profile, motile, or planktonic), and life form (including unicellular, filamentous, or colonial life; and weak, medium, or strong attachment) (Table S2). These trait assignments were compiled based on established descriptions in Passy (2007a), Witteveen et al. (2020), and Wu et al. (2017). For the key trait of cell size, which affects growth rate, metabolism, and resource acquisition in both benthic and phytoplanktonic algae, we applied the classification criteria from Passy (2007b) and Kruk et al. (2010).

### 2.5 Data Analysis

To estimate the taxonomic nestedness of algal assemblages across the entire study area, we calculated the nestedness value using the NODF metric in the NODF2.0 software (Almeida‐Neto et al. 2008). This software sorts the matrix based on the number of occurrences in each row and draws a diagonal line between species presence and absence (Guimaraesjr and Guimaraes 2006). Then, the Gower distance in the *vegan* package of R was employed to calculate the similarity of species traits, and the UPGMA clustering algorithm in the *ape* package was used to convert this into a functional dendrogram (Paradis and Schliep 2019). Using the “*treeNodfTest*” function in the *CommEcol* package and the “*permRows*” null model, the species occurrence frequency matrix sorted by environmental factors was associated with the functional dendrogram to calculate traitNODF (Melo et al. 2014). Finally, the matrix was rearranged through 999 permutation repetitions to assess whether the observed traitNODF and its components differed from random expectations (Matthews et al. 2015). The phyloNODF was calculated using the same method, where the taxonomic names of species were used to construct a phylogenetic tree, which was then associated with the species occurrence matrix. Additionally, this study tested whether species with specific traits exhibited nestedness in environmental conditions. By generating a tree based on environmental factors and sorting the species presence matrix according to species traits, envNODF was calculated using the same approach.

## 3 Results

### 3.1 Taxonomic Nestedness of Algal Assemblages

**FIGURE 2:**
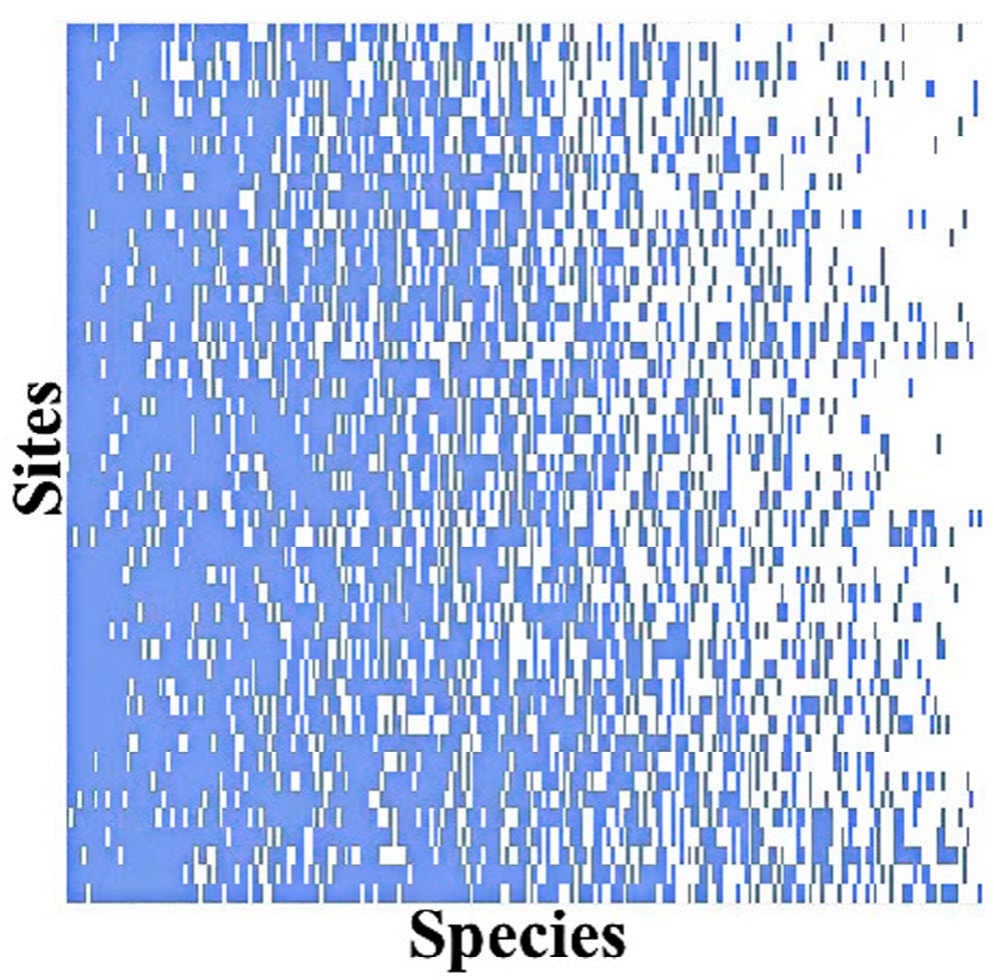
Maximally nested presence–absence matrix of species across all sampling sites in Oujiang river basin.

The species‐site matrix across the entire watershed exhibited a high and statistically significant degree of nestedness (NODF = 70.01, *p* < 0.001) (Figure 2, Table 1). Furthermore, both the sites (NODFc = 69.86, *p* < 0.001) and the species (NODFr = 70.02, *p* < 0.001) demonstrated a highly nested pattern. The NODF values for S1, S2, S3, and S4 all indicated significant nestedness across the four types of river sections. Among these, S1 exhibited the lowest NODF and NODFr values, while S2 had the lowest NODFc.

**TABLE 1:** Nestedness for the whole catchment and for different river sections in Oujiang river basin.

	Metric	Observed NODF	Simulated NODF	*p*
ALL	NODF	70.01	48.25	< 0.001
	NODFc	69.86	46.77	< 0.001
	NODFr	70.02	48.31	< 0.001
S1	NODF	50.63	35.02	< 0.001
	NODFc	71.28	44.78	< 0.001
	NODFr	50.61	35.01	< 0.001
S2	NODF	65.41	45.41	< 0.001
	NODFc	65.55	45.11	< 0.001
	NODFr	65.41	45.41	< 0.001
S3	NODF	66.71	45.45	< 0.001
	NODFc	73.33	47.62	< 0.001
	NODFr	66.69	45.45	< 0.001
S4	NODF	69.35	47.49	< 0.001
	NODFc	71.12	47.35	< 0.001
	NODFr	69.34	47.49	< 0.001

### 3.2 Response of traitNODF to Environmental Factors

**FIGURE 3:**
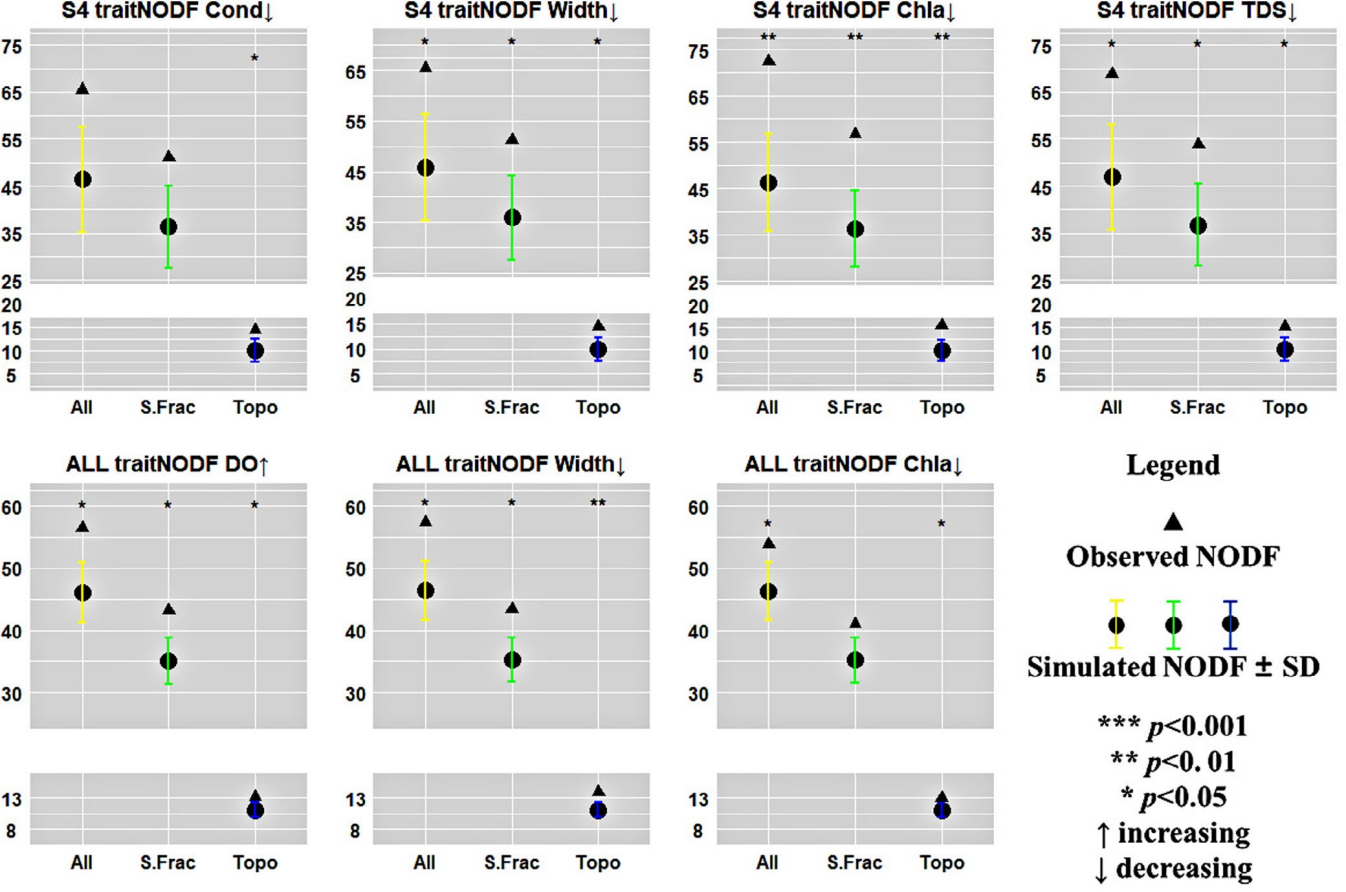
Response of traitNODF to environmental factors in the Oujiang river basin. ALL, entire watershed; All, treeNODF; Chl*a*, chlorophyll‐*a*; Cond, conductivity; DO, dissolved oxygen; S.Frac, S.Fraction; TDS, total dissolved solids; Topo, topoNODF.

The results of the traitNODF analysis (Figure 3) (based on permutation tests for significance) indicated that, when sites were sorted by Cond, the observed traitNODF in S4 was significantly higher than random expectations and was primarily driven by the topoNODF (*p* < 0.05). This suggests a non‐random filtering of functional traits under low conductivity conditions. Furthermore, a significant increase in traitNODF was consistently observed in S4 under narrower river widths (*p* < 0.05), lower Chl*a* (*p* < 0.01), and TDS (*p* < 0.05), with topoNODF remaining the dominant driver across these environmental gradients. Similarly, at the entire watershed, traitNODF became significantly more pronounced when river widths decreased (*p* < 0.05) or DO increased (*p* < 0.05).

### 3.3 Response of phyloNODF to Environmental Factors

**FIGURE 4:**
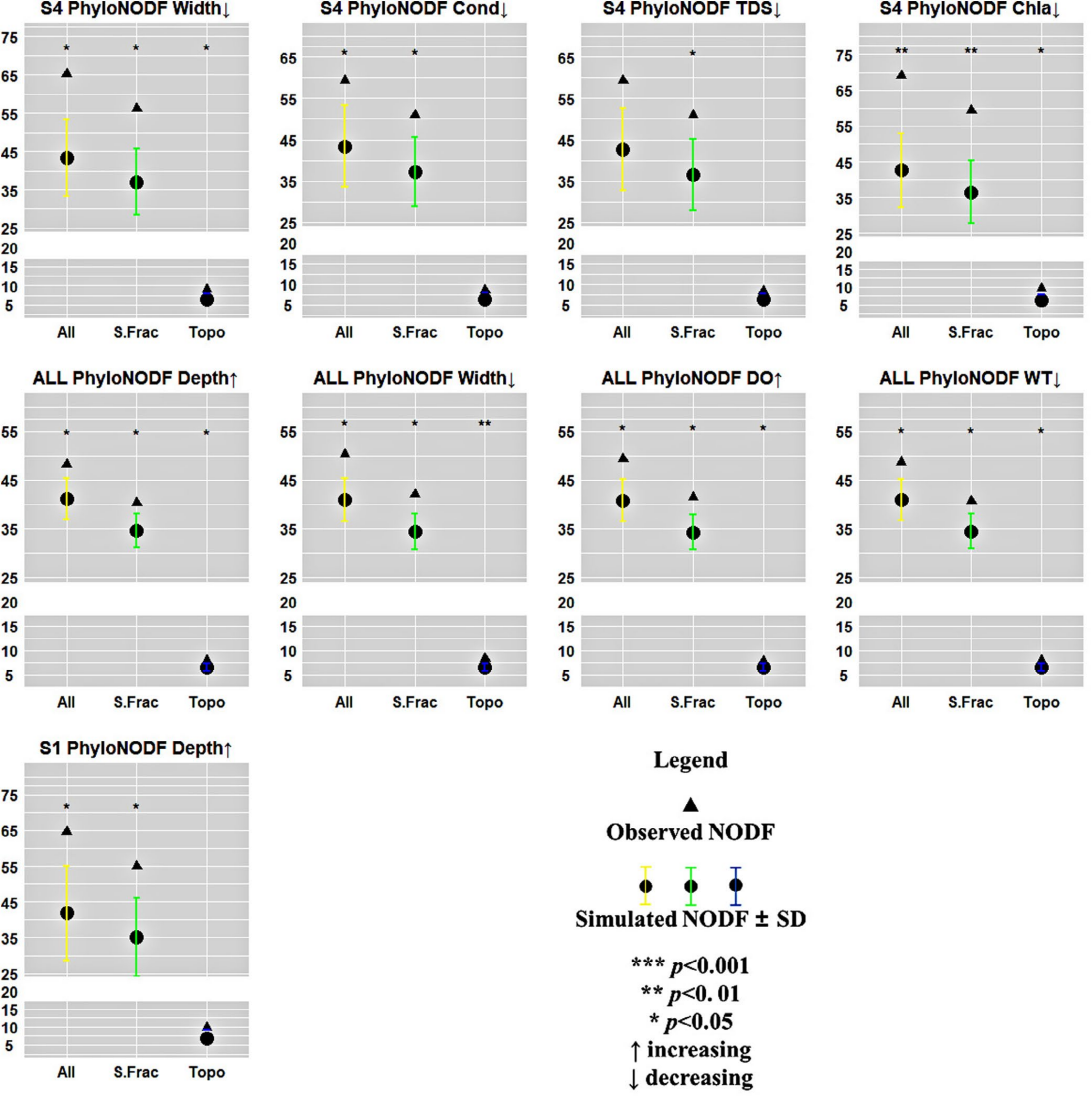
Response of phyloNODF to environmental factors in the Oujiang river basin. The abbreviations are as in Figure 3.

The results of phyloNODF (Figure 4) indicated that, based on the analysis sorted by increasing depth, the observed phyloNODF for S1 and the entire watershed were significantly higher than random expectations (*p* < 0.05), while greater depths appeared to support more complex phylogenetic relationships. For the entire watershed, both S.Fraction and topoNODF were significantly higher than expected, and the nestedness was more strongly correlated with S.Fraction (*p* < 0.05). Moreover, phyloNODF increased significantly with narrow widths (*p* < 0.01), with topoNODF remaining the dominant driver across these environmental gradients. In S4, stronger phyloNODF was observed under narrower widths (*p* < 0.05), lower Cond (*p* < 0.05), and lower Chl*a* (*p* < 0.01), with both S.Fraction and topoNODF as significant drivers.

### 3.4 Response of envNODF to Species Traits

**FIGURE 5:**
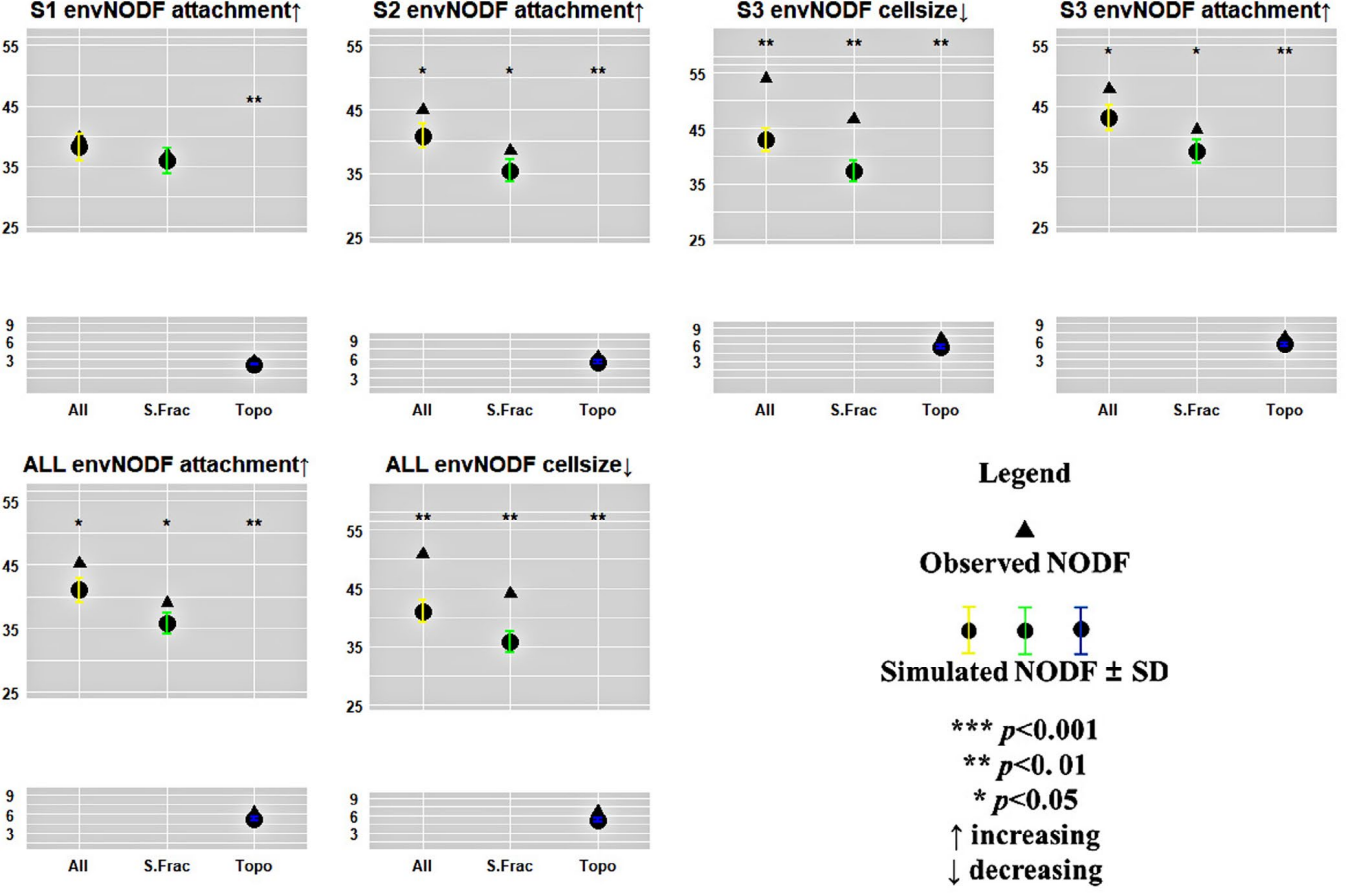
Response of envNODF to species traits in Oujiang river basin. The abbreviations are as in Figure 3.

The results of envNODF (Figure 5) indicated that, based on the analysis sorted by increasing species attachment capability, the observed envNODF for S2 (*p* < 0.05), S3 (*p* < 0.05), and the entire watershed (*p* < 0.05) were significantly higher than random expectations. Both S.Fraction (*p* < 0.05) and topoNODF (*p* < 0.01) were significantly higher than expected, and nestedness in S1, S2, S3, and the entire watershed was more strongly correlated with the topoNODF. In S2, S3, and the entire watershed, higher algal attachment capability corresponded with more pronounced envNODF, while species with lower attachment occupied more complex habitats across sampling sites.

When analyzed based on decreasing species cell size, the observed envNODF for S3 (*p* < 0.01) and the entire watershed (*p* < 0.01) were significantly higher than random expectations. Additionally, both S.Fraction (*p* < 0.01) and topoNODF (*p* < 0.01) were significantly higher than expected, and nestedness was more closely related to the topoNODF. Smaller species cell sizes in S3 and the entire watershed were associated with more evident envNODF, while larger species tended to occupy more complex habitats among sampling sites.

## 4 Discussion

This study revealed that algal communities in rivers affected by SHPs exhibited significant nestedness at the taxonomic, phylogenetic, and functional levels. Currently, research on nestedness has primarily focused on island systems, including land‐bridge islands, oceanic islands, and fragmented natural habitats (such as forest patches, urban parks, and mountain floras) (Cook and Quinn 1998; Tan et al. 2021; Wang et al. 2013; Watling and Donnelly 2006; Worthen et al. 1996; Wright et al. 1998), and even continental continuous habitat systems (Hylander et al. 2005; Patterson et al. 1996; Worthen et al. 1996). However, systematic studies on algal nestedness in river ecosystems affected by SHPs have been relatively scarce. Therefore, this study fills this research gap by exploring the nestedness of algae, providing a theoretical basis for the protection and management of river ecosystems.

The study found significant nestedness across the entire watershed and different river sections. Among them, the NODFr in the S1 was lower than that of the other river sections. Ecologically, this indicates that upstream headwaters, being in a more natural state with minimal SHP influence, fostered higher species turnover and greater community stochasticity (Qi, Lin, et al. 2024). This is consistent with our trait observations that S1 harbored more low‐profile algae adapted to faster‐flowing, nutrient‐efficient conditions (Passy 2007b). The resulting lower nestedness suggests a greater potential for these reaches to act as a diverse species pool, which is crucial for ecosystem resilience (Wang, Wu, Tang, Zhou, and Cai 2022). Due to the SHPs‐induced reservoir section, a sharp decrease of DO from S1 to S2, which in turn affected the release of organic pollutants from the reservoir substrate, results in a lower NODFc in the reservoir sections compared to the other sections, indicating greater variation between sampling sites in the reservoir section (Kunz et al. 2011). Subsequently, differences in environmental variables led to differences in community composition of riverine algae in different river sections (Lopez‐Delgado et al. 2020).

Environmental factors drove the restructuring of algal functional composition, which in turn shaped the observed increase in traitNODF. The lower functional redundancy observed in S4 of SHP‐affected rivers in our related research supports this interpretation (Chua et al. 2019). These results supported the important role of environmental filtering in shaping community functional structure (Chen et al. 2022). Specific environmental constraints, such as narrow width and low nutrient levels, enhance the nestedness of algal traits by filtering out particular trait combinations. In contrast, the wider, nutrient‐rich reservoir sections host richer trait spectra with lower nestedness, suggesting they should be conservation priorities due to their greater vulnerability to species invasion and functional homogenization (Qi, Lin, et al. 2024). The findings of phyloNODF suggested that environmental conditions shape the phylogenetic structure of communities by influencing the phylogenetic relationships among species (Matthews et al. 2015). While SHPs enhance the nested structure of algal communities in S4 through environmental filtering, phyloNODF was lower in S2, and the non‐random loss of species may constrain the adaptive potential of algal communities to future environmental changes (Cadotte et al. 2017).

The development of SHPs had altered the hydrological and physicochemical indices of different river sections also promoting different habitat conditions, and species respond rapidly to such environmental changes through their traits (Riato et al. 2017). The findings revealed the important role of species traits in environmental filtering and niche differentiation (McAbendroth et al. 2005; Melo et al. 2014). In particular, the presence of special species (such as those with broad ecological tolerance and good dispersibility) may further enhance envNODF (Atmar and Patterson 1993). The enhanced envNODF observed in the entire watershed and S3 with smaller algal cell size can be attributed to the stronger recovery capacity associated with higher growth rates in smaller algae (Litchman and Klausmeier 2008). The substrate parameters and the riverbank characteristics were less affected in S3, which was replenished by ecological flows (Qi, Lin, et al. 2024). Our study found that the ecosystem in S3, likely stabilized by ecological flow supplementation, contained many larger cell size algae (Jacobucci et al. 2011; Rimet and Bouchez 2012; Wu et al. 2017). Therefore, when riverine algae cell size is smaller, the species are more likely to form nestedness in Oujiang river basin.

Although significant nestedness was observed in both species' composition and the topologies of trait and phylogenetic structures, the topoNODF typically accounted for only a small portion of the treeNODF values. Likewise, other related studies, such as birds (Matthews et al. 2015) and amphibians (Chen et al. 2022), also found a lower contribution of topoNODF, but the significant contribution of topoNODF components remains important as they reflect the high nestedness of functional and phylogenetic topologies in watersheds affected by SHPs. Additionally, the nestedness of sampling sites in S2 was relatively low, which can be attributed to the greater diversity of species traits in S2, significant differences in environmental variables (such as velocity and width) compared to other river sections, and variations in sampling sites due to the construction of SHPs (Wang, Wu, Tang, Wang, and Cai 2022). Similarly, the differences between sampling sites in S3 were influenced by variations in ecological flow (Qi, Lin, et al. 2024).

The presence of nested patterns is generally considered beneficial for the maintenance of species diversity and the enhancement of ecosystem stability (Tornés and Ruhí 2013). This study found that under the influence of SHPs with ecological flow, the river algal communities exhibited significant nestedness, which may help buffer the impacts of environmental changes on community structure. However, the enhancement of nestedness could also lead to increased sensitivity of communities to environmental changes, especially when there are abrupt shifts in environmental conditions (Matthews et al. 2015). Thus, the fact that this study is based on a single, one‐time sampling event represents a critical limitation. As seasonal hydrological variations, particularly during dry periods, can drastically alter habitat conditions and ecological function (Martínez et al. 2013; McIntosh et al. 2002), our conclusions require validation through multi‐seasonal and long‐term monitoring. Additionally, the limited availability of detailed hydrological and morphological data for some reservoirs, especially those under private management, where on‐site measurements and public data access were restricted further constrained our ability to fully characterize habitat conditions and their ecological impacts. More critically, this data gap was compounded by inconsistencies between the officially mandated ecological flow and the actual discharges observed at many privately operated SHPs, which prevented a more rigorous quantitative linkage between specific flow rates and the ecological patterns we documented.

Therefore, future research should be expanded to detail the impacts of diverse SHP types on hydraulic conditions and fluvial geomorphology across different geographical river sections (Fencl et al. 2015). These findings have important implications for the management of SHPs and the protection of river ecosystems. Given our observation that some SHPs failed to release mandated ecological flows, strengthening compliance monitoring is imperative (Lin et al. 2024). Implementing a mix of compensatory and punitive measures could incentivize private SHP operators to adhere to ecological flow regulations (Zhang et al. 2021). In river basins like the Oujiang with cascading SHPs, management must shift from evaluating single dams to conducting cumulative impact assessments to understand their aggregate effect on river connectivity and ecosystem integrity (Athayde et al. 2019; Xiao et al. 2019). Furthermore, protecting idiosyncratic species with unique functional traits or phylogenetic relationships is crucial for maintaining the nestedness of communities and the functionality of ecosystems. Based on the varying nestedness patterns and functional redundancy across river segments (e.g., reservoir S2, dewatering reach S3), we recommend implementing differentiated ecological flow‐release strategies to mitigate excessive environmental filtering of biodiversity.

## 5 Conclusion

This study reveals that SHPs with ecological flows significantly induce nestedness in riverine algal communities across taxonomic, functional, and phylogenetic dimensions, primarily mediated through environmental filtering. The stability of functional and phylogenetic nestedness in the reservoir and dewatering sections, despite environmental fluctuations, underscores their heightened vulnerability and altered ecological state. Species with weaker attachment abilities and larger cell sizes tend to inhabit unique habitats within the watershed, primarily in the reservoir, suggesting that the construction of SHPs has significantly altered reservoir habitats. These findings provide new insights into the ecological impacts of SHPs and offer scientific evidence for river ecosystem conservation and management. To translate this evidence into effective action, policymakers should design and enforce adaptive ecological flow releases that are explicitly informed by local community traits and phylogenetic structures. Future efforts should prioritize long‐term, multi‐seasonal monitoring to unravel the dynamics between nestedness and ecosystem stability, and to develop quantitative flow‐ecological response frameworks. Ultimately, integrating such ecological insights into policy and governance is pivotal for achieving the dual goals of renewable energy development and the conservation of aquatic biodiversity and ecosystem resilience.

## Author Contributions

**Xinxin Qi:** conceptualization (equal), data curation (equal), formal analysis (equal), investigation (equal), methodology (equal), software (equal), visualization (equal), writing – original draft (equal). **Zongwei Lin:** investigation (equal), writing – original draft (supporting), writing – review and editing (equal). **Yuting Wang:** investigation (equal), writing – original draft (supporting), writing – review and editing (equal). **Yuke Duan:** investigation (equal), writing – original draft (supporting), writing – review and editing (equal). **Jiuli Shi:** writing – original draft (supporting), writing – review and editing (equal). **Huimin Gao:** writing – original draft (supporting), writing – review and editing (equal). **Sangar Khan:** investigation (equal), writing – original draft (supporting), writing – review and editing (equal). **Naicheng Wu:** conceptualization (lead), data curation (equal), formal analysis (equal), funding acquisition (lead), investigation (equal), project administration (lead), software (equal), supervision (lead), writing – original draft (supporting), writing – review and editing (lead).

## Conflicts of Interest

The authors declare no conflicts of interest.

## Supporting information


**Table S1:** Characteristics of the 15 surveyed SHPs in the Oujiang river basin. The abbreviations of each SHP are listed in parentheses.
**Table S2:** Categories of riverine algae traits in Oujiang river basin.

## Data Availability

The data that support the findings of this study are available in the Supporting Information.
