# Supplementary material for: Small Hydropower Plants With Ecological Flow Influence Nestedness of Riverine Algae: Insights From treeNODF Analysis in Oujiang River Basin
Source: Ecol Evol. 2026 Jan 12;16(1):e72930. doi: 10.1002/ece3.72930 (PMC12793786; doi:10.1002/ece3.72930)
Supplement: Supplementary file 1 — Table S1: Characteristics of the 15 surveyed SHPs in the Oujiang river basin. The abbreviations of each SHP are listed in parentheses. Table S2: Categories of riverine algae traits in Oujiang river basin. [file ECE3-16-e72930-s001.docx]

SUPPORTING INFORMATION

**Small hydropower plants with ecological flow influence nestedness of riverine algae: insights from treeNODF analysis in Oujiang river basin**

Xinxin Qi^1,2,3^, Zongwei Lin^1,3,4^, Yuting Wang^1,3^, Yuke Duan^1,3^, Jiuli Shi^1,3^, Huimin Gao^1,3^, Sangar Khan^1,3^, Naicheng Wu^1,3,5^*

^1^ Department of Geography and Spatial Information Techniques, Ningbo University, Ningbo 315211, China

^2^ Lianyungang Hean Middle School, Lianyungang 222100, China

^3^ Zhejiang-Germany Joint Laboratory on Remote Sensing of Coastal Ecosystem, Ningbo University, Ningbo, China

^4^ Department of Geosciences and Geography, University of Helsinki, Helsinki, Finland

^5^ Department of Hydrology and Water Resources, University of Kiel, Kiel, Germany

^*^ Corresponding author: Naicheng Wu ([naichengwu88@gmail.com](mailto:naichengwu88@gmail.com); wunaicheng@nbu.edu.cn)

**Table S1** Characteristics of the 15 surveyed SHPs in the Oujiang river basin. The abbreviations of each SHP are listed in parentheses.

| River | Name | Type | Reservoir depth (m) | Ecological flow (m^3^/s) |
| --- | --- | --- | --- | --- |
| Panxi river | Panxi first class hydropower station (PXI) | Non-diversion with storage |  | 0.074 |
|  | Tianshengqiao hydropower station (TSQ) | Diversion with storage |  | 0.093 |
|  | Panxi fifth class hydropower station (PXV) | Diversion with storage |  |  |
|  | Panxi sixth class hydropower station (PXVI) | Diversion with storage |  | 0.070 |
| Xiaoanxi river | Sanqiao hydropower station (SQ) | Diversion with storage |  | 0.222 |
|  | Santan hydropower station (ST) | Diversion with storage | 4.330 | 0.28 |
|  | Lingxi hydropower station (LX) | Diversion with storage |  |  |
|  | Yuantan hydropower station (YT) | Diversion with storage |  | 0.6 |
| Songyinxi river | Shimapu hydropower station (SMP) | Non-diversion with storage |  | 2.690 |
|  | Yuxi hydropower station (YX) | Non-diversion with storage |  | 3.350 |
|  | Hexi hydropower station (HX) | Non-diversion with storage |  | 3.400 |
| Longquanxi river | Yunzhang hydropower station (YZ) | Diversion with storage | 0.690 |  |
| Xiaoxi river | Longshitan hydropower station (LST) | Diversion with storage |  |  |
|  | Huangkengxu first class hydropower station (HKXI) | Diversion with storage | 0.410 | 0.054 |
|  | Huangkengxu second class hydropower station (HKXII) | Diversion with storage | 0.550 |  |

**Table S2** Categories of riverine algae traits in Oujiang river basin.

| Traits | Categories | Codes |
| --- | --- | --- |
| Cell size | Nano (0–100 µm^3^) | CellSize01 |
|  | Micro (100–300 µm^3^) | CellSize02 |
|  | Meso (300–600 µm^3^) | CellSize03 |
|  | Macro (600–1,500 µm^3^) | CellSize04 |
|  | Large (≥ 1,500 µm^3^) | CellSize05 |
| Guild | Low profile | LowPro |
|  | High profile | HigPro |
|  | Motile taxa | MotTax |
|  | Planktonic taxa | PlaTax |
| Life form | Unicellular life form | LF_Uni |
|  | Filamentous life form | LF_Fil |
|  | Colonial life form | LF_Col |
|  | Low attachment | ATT_Low |
|  | Medium attachment | ATT_Med |
|  | High attachment | ATT_Hig |
